# Virgin Olive Oil Quality Is Affected by the Microbiota that Comprise the Biotic Fraction of the Oil

**DOI:** 10.3390/microorganisms8050663

**Published:** 2020-05-01

**Authors:** Biagi Angelo Zullo, Gino Ciafardini

**Affiliations:** Department of Agricultural, Environmental and Food Sciences, University of Molise, Via De Sanctis, I-86100 Campobasso, Italy; ciafardi@unimol.it

**Keywords:** virgin olive oil (VOO), biotic fraction, yeast species, virgin olive oil quality, technological and health properties

## Abstract

This review summarizes the current knowledge on the effects of oil-borne yeasts on the physicochemical, sensorial, and health-related characteristics of virgin olive oil (VOO) during storage. Bacteria, yeasts, and molds constitute the biotic fraction of freshly produced VOO. During storage, the bacteria and molds often die after a short period, while the yeasts survive and condition the quality of VOO. To date, approximately twenty-four yeast species have been isolated from different types of olive oil and its by-products, and seven of these species have been identified as new species. The activity of some yeasts of the biotic fraction of olive oil improves the sensorial characteristics of VOO. Some yeasts can also worsen the quality of the product by allowing the appearance of defects, oxidation of polar phenols, and triacylglycerol hydrolysis. Some yeast species of VOO show *in vitro* beneficial health effects, such as probiotic and antioxidant activities.

## 1. Introduction

Virgin olive oil (VOO) is a product obtained by mechanical extraction from the olive fruit and can be consumed without further refining. It is the most important vegetable oil used for human nutrition in the Mediterranean area. It is known worldwide for health benefits, which are attributed to its antioxidant component and high content of unsaturated fatty acids [1,2]. Freshly produced VOO appears cloudy due to the presence of micro-drops of oil-mill wastewater and numerous solid particles of olive skin and pulp covered by water films, representing the suspended fraction of VOO [3,4]. During storage, the suspended material settles at the bottom of the container to form a sediment. To reduce the suspended material in oil, freshly produced VOO is filtered through cotton filters under pressure in the bottling industry. However, filtration of the newly produced VOO has not been completely accepted, because some studies have shown that filtration reduces the oil stability and the concentration of phenolic compounds during storage [5,6]. On the contrary, other authors have reported that elimination of the sediment improves the shelf life of VOO and prevents the development of an off flavor during storage [7,8]. The chemical composition of VOO is represented by major compounds (98% of the total oil weight) and minor compounds (about 2%), including more than 230 chemical compounds, such as aliphatic and triterpenic alcohols, sterols, hydrocarbons, volatile compounds, and antioxidants. The major antioxidants of VOO include carotenes and bioactive phenolic compounds (apolar and polar phenols). Apolar phenols such as tocopherols are also found in other vegetable oils, while the polar phenolic component is typical of VOO [9]. The polar phenolic compounds of VOO include phenolic acids and derivatives, phenolic alcohols, secoiridoids, lignans, and flavonoids, which exhibit antioxidant, anti-inflammatory, anticancer, antimicrobial, and antiviral activities [10,11,12,13,14,15,16]. Since VOO is produced over a short period of time, it must be stored for the rest of the year until the next olive oil campaign. During storage, VOO is subject to hydrolysis, oxidation, autoxidation, and polymerization, leading to the deterioration of its components, quality and nutritional values, alteration of its oxidative stability, and reduction in its health benefits [17,18]. In a previous investigation, Ciafardini and Zullo [19] demonstrated, for the first time, the presence of a biotic fraction, consisting of the microbiota, in the oily mass of the newly produced olive oil. The microbiota of VOO includes yeasts, bacteria, and molds. Several studies conducted on the VOO microbiota showed that the yeasts were capable of conditioning the physicochemical, sensorial, and health-related characteristics of VOO during storage [20,21,22,23,24]. In this study, the effects of the biotic fraction of VOO on product quality during its storage have been reviewed, and the possible biotechnological exploitation of the health benefits of some oil-borne yeasts has been discussed.

## 2. The Microbiota of VOO

The oily fraction of healthy olive fruits is free of microorganisms before harvest. However, during the extraction phase in the mill, the oily fraction is colonized by microorganisms from various sources, including the carposphere of the olives [25,26]. The bacteria and molds present in the oily mass of the newly produced VOO often die after a short time, while the yeasts survive and constitute the microbiota of freshly produced VOO [25,26,27].

### 2.1. Yeast Survival and Distribution

**Figure 1:**
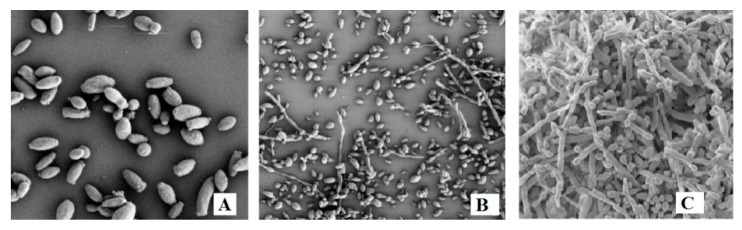
SEM observation of the *Yamadazyma terventina* 2092 yeast strain after three months of incubation in virgin olive oil (VOO) with low (**A**) and high (**B**) total polar phenolic content; (**C**) Yeast growth in the CMA medium.

The concentration of yeasts detected in VOO depends on several factors, such as filtration, sedimentation, and the physicochemical composition. Filtration reduces the initial microbial concentration of VOO, which, in some cases, can be partially or completely restored by cell replication during storage. Although filtered oils contain fewer yeast species, some of them demonstrate the maximum colony forming units (CFUs) number of yeasts, ranging from 3.50 logs to 4.20 logs per mL [28,29]. Similarly, in unfiltered olive oils, the maximum CFUs number of yeasts ranged from 4.56 logs per mL in the newly produced olive oils and 3.03 logs per mL after one year of storage [30]. The survival of yeasts in the oily mass is conditioned by the water content and chemical composition of VOO [29,31,32]. The water content in VOOs varies from 0.15% (w/w) to over 0.36% (w/w), depending on the use of the oil extraction system and product filtration. However, water content above 0.25% (w/w) is considered high, because this not only promotes the growth of microorganisms, but also activates enzymes that are harmful to oil quality [33]. Depending on the concentration of the total polar phenolic compounds in VOO during storage, the physiological activity of yeasts is modified, exerting a strong selective pressure on the survival of yeast species. A study was performed with the *Yamadazyma terventina* 2092 dimorphic yeast strain, which was inoculated into VOO with a low or high phenolic compound content. Dimorphic yeasts demonstrate an equilibrium between spherical and polarized growth (pseudohyphal tip elongation), and this can be triggered while transitioning from favorable to unfavorable environmental conditions. In the previous study, the *Y. terventina* 2092 yeast strain was inoculated into VOO with different phenolic compound concentrations (100 mg and 350 mg caffeic acid equivalent (CAE) per kg) and stored at 15 °C for three months. Corn Meal Agar medium (CMA, Oxoid code CM 0103) was used for pseudohyphae production of *Yamadazyma terventina* 2092 dimorphic yeast strain. The yeast cells were extracted from the oil samples at the end of the incubation period and analyzed with a scanning electron microscope (SEM) according to Zullo et al. [29] The *Y. terventina* 2092 dimorphic yeast strain showed polarized growth only in the sample of VOO with a high total polar phenolic content equal to 350 mg CAE per kg (Figure 1).

In a previous research study, it was observed that a high phenolic content shortens the survival period in olive oil of some opportunistic pathogenic yeasts, including *Candida parapsilosis* [31]. The fatty acids and triglycerides present in VOO also inhibit the growth of yeasts. Several yeast species, such as *Meyerozyma guilliermondii, C. parapsilosis,* and *Candida diddensiae*, have been reported to exhibit concentration-dependent sensitivity to linoleic acid [29]. The survival of yeasts during oil storage is also affected by the blending of monovarietal VOOs. A recent study conducted by Zullo and Ciafardini [34] has shown that the number of yeasts markedly decreased in blended VOO during the storage compared to the starting monovarietal VOOs. In the oleic habitat, basidiomycetous yeasts (*Cryptococcus*, *Rhodotorula*, and *Sporobolus*) were found on the leaves and on the fruits and not in the freshly produced VOO [35]. Oil-borne yeasts have been isolated from oils extracted from different varieties of olives, using the method described by Zullo and Ciafardini [29]. In detail, 10 mL of the oil samples were micro-filtered through sterile nitrocellulose filters, with a porosity of 0.45 µm. The nitrocellulose filter of the sample was homogenized with a Turrax homogenizer (IKA, Milan, Italy), in the presence of sterile physiological solution. Then, the initial volume equal to 10 mL was reconstituted and the microbial suspension was used for 10-fold serial dilutions, with a sterile physiological solution of 0.9% (w/v) NaCl. Yeasts were detected using Petri dishes with MYGP agar medium, as described by Ciafardini and Zullo [21]. Several yeast species were isolated from different types of olive oils, and seven of these were new species (Table 1). Yeasts belonging to *C. diddensiae* and *Nakazawaea wickerhamii* have been found in commercial VOO, available in supermarkets, and olive oil produced from the Moraiolo and Frantoio varieties [23,28]. Yeast species such as *Candida norvegica*, *Candida oleophila*, *Debariomyces hansenii*, *C. diddensiae*, and *Wicherhamomyces anomalus* were found in the newly produced Taggiasca olive oil, while only *W. anomalus* survived in the same oil after six months of storage [26]. Other species including *Groenewaldozyma aurigiensis*, and *Lachancea fermentati* were isolated from olive oil produced in Spain [36], while the yeast *Kuraishia capsulata* was isolated by us for the first time in a blended VOO, prepared from an oil of an Italian origin and one from Portugal. *K. capsulata* is often recovered from frass or tunnels of larvae underneath the bark of certain conifers. This yeast species produces extracellular polysaccharides, allowing the cells to adhere to the bark beetles, and this adhesive property probably aids in dispersal [37]. A low cell count of opportunistic pathogenic yeast species, such as *C. parapsilosis* and *M. guilliermondii*, isolated from commercial olive oil, was observed [29]. In the sediments of VOO and spoiled olive oil, unknown yeast species including *Brettanomyces acidodurans*, *Candida adriatica*, *Kuraishia mediterranea, Nakazawaea molendinolei, Ogateae histrianica, Ogateae kolombanensis,* and *Y. terventina* were recently isolated and classified as new species [36,38,39,40,41]. *C. adriatica*, *N. molendinolei*, and *Y. terventina* are yeast species that are frequently isolated from Italian oils produced in central and northern Italy. Other species, such as *K. mediterranea*, *O. histrianica*, and *O. kolombanensis*, are methylotrophic yeast species that are frequently isolated from olive oil sediments. These yeast species can assimilate methanol, produced as a by-product of pectin metabolism, because they are pectinolytic [39,41]. Pectinolytic yeasts use polygalacturonase to utilize pectin for growth and have been previously identified as the causative agents of spoilage defects in table olives [42,43]. Similarly, the new yeast *B. acidodurans* produces acetic acid in olive oil sediments, resulting in a wine-vinegary defect in the product [36]. Other yeast species, including *Saccharomyces cerevisiae, Yamadazyma mexicana*, *Yamadazyma nakazawaea*, and *Candida* spp. (three species), were isolated from Sardinian olive oils. *C. adriatica* was the only yeast species identified in VOO obtained from olives of the Semidana variety. From VOO of the Nera di Gonnos variety, three yeast species, namely *S. cerevisiae*, *Candida temnochilae*, and *Y. nakazawaea*, were obtained, while from VOO of the Nocellara del Belice variety, two yeast species, *Y. mexicana* and *Candida dendronema*, were obtained [44].

### 2.2. Bacteria and Molds

Bacterial species, such as *Stenotrophomonas rhizophila*, *Pseudomonas cedrina*, *Pseudomonas stutzeri*, and *Pantoea septica*, were found in the one-year stored olive oils obtained from blends of five different olive cultivars, including Leccino, Coratina, Ogliarola, Frantoio, and Cellina di Nardò varieties. Two bacterial strains of the species *P. septica* produce carotenoids and bioemulsifiers, enabling the bacteria to survive and grow an unfavorable substrate [45]. Other bacteria, belonging to *Bacillus* spp., *Brevibacillus* spp., *Micrococcus* spp., *Staphylococcus* spp., *Kocuria* spp., *Lysinbacillus* spp., and *Lactobacillus* spp., were found in VOOs, which were subject to enrichment and obtained from different Italian varieties [46]. A study conducted by Zullo et al. [27] showed that coliform bacteria could survive and reproduce in VOO containing low levels of total polar phenols. The laboratory inoculation trials demonstrated that when the bacterium *Escherichia coli*, isolated from the carposheres of olives, was transferred to olive oil containing high polar phenol content, its growth was completely inhibited after 15 days of storage. On the contrary, the bacterium reproduced quickly when it was inoculated in VOO containing a lower concentration of polar phenols. To date, a few studies on the presence of bacteria in VOO have focused on biodiversity and their potential biotechnological utility. However, there is a lack of studies on the influence of bacteria on the VOO quality during storage. The mold content in VOO obtained from healthy olives was reported to be low because the mold died during storage [30]. Molds, from different VOO samples, mainly belonged to the genus *Aspergillus* [47].

## 3. Influence of Yeasts on VOO Quality

Based on enzymatic activities, microorganisms of the olive carphosheres can influence the oil quality during the extraction process in the mill. In a study by Vichi et al. [48], it was reported that the oils obtained from microbiologically-contaminated olives were of poor quality and the effect of the microbiota on oil characteristics was greater than that exerted by malaxation conditions, such as time and temperature. Some yeasts present in a newly unfiltered VOO can remain viable and metabolically active during the storage of the oil, and according to their metabolic capacities, they can improve or worsen the physicochemical and sensory characteristics of VOO [19,20,49]. Enzymes produced by yeasts that are isolated from olives or olive oil include β-glucosidase, β-glucanase, phenoloxidase, peroxidase, lipase, esterase, and cellulase [19,21,23,44,49,50,51]. Moreover, β-glucosidase and esterase act on the bitter glucoside oleuropein and its derivative oleuropein aglycone, respectively, in olive oil [19]. The enzymatic hydrolysis of oleuropein reduces the bitter taste and improves the antioxidant and scavenging activities of VOO [19]. Other enzymes, such as lipase, phenoloxidase, and peroxidase, deteriorate the oil quality [52,53,54,55]. During the storage of VOO, some yeast species produce lipase (glycerol-ester-hydrolase, E.C. 3.1.1.3) that hydrolyzes the fatty acid acyl ester bonds of acylglycerols, consequently increasing the content of free fatty acids, which are very sensitive to autoxidation in the oil [50,56]. In detail, laboratory experiments with the inoculation of two oil-borne lipase positive yeast species, *C. adriatica* 1985 and *C. parapsilosis* 1984 yeast strains, and the lipase-negative *Candida boidinii* 1638 yeast strain, showed that the lipase-positive yeast strains increased the free fatty acid content and consequently reduced the quality during storage [21,56,57,58]. The lipase activity of yeasts is influenced by the ratio of the aqueous and organic phases, and it reaches the maximum value when the water added to the oil is 1% for *C. adriatica* and 0.25% for *C. parapsilosis* [21,50]. The polar phenol content of olive oil also influences the viability and lipolytic activity of the lipase-producing yeasts. Laboratory experiments conducted with olive oil with increasing contents of total polar phenols (89 mg, 159 mg and 540 mg CAE per kg) determined the percentages of lipase-producing yeasts that could hydrolyze triacylglycerol (100%, 67%, and 11%, respectively) [49]. During the storage of an unfiltered VOO, some yeast strains of the microbiota can influence the sensory characteristics of VOO, by reducing positive attributes such as fruitiness, bitterness, and pungency, or allowing the appearance of unpleasant sensory notes, which are classified into four groups: “fusty,” “musty,” “winey-vinegary,” and “rancid”, according to the current olive oil regulations [59]. Oleuropein and its aglycon form, among the polar phenols, are responsible for the bitterness of VOOs [60]. Oleacein and oleocanthal are responsible for the pungency of certain olive oils [61]. These positive attributes are not the criteria for an olive oil classification. Guerrini et al. [62] showed that sensory defects and specific volatile compounds (2-butanone, butyric acid, 2-heptanol, octanoic acid, and 1-octen-3-ol) were related to both yeast and mold concentrations in the freshly extracted and filtered oils. Inoculation trials conducted with the Leccino VOO demonstrated that during storage, some oil-borne yeast strains were responsible for the appearance of sensory defects. In detail, a micro-filtered Leccino VOO was inoculated with six oil-borne yeast strains belonging to *C. adriatica*, *C. diddensiae*, and *N. wickerhamii*. After four months of storage, sensory defects, such as “muddy-sediment”, “rancid” or both, were found in olive oil treated with the *C. adriatica* 1933, *N. wicherhamii* 1885, and *C. diddensiae* 1912 and 1913 strains. In contrast, olive oil samples treated with the *C. diddensiae* 1918 and 1922 strains were defect-free and remained commercially classified as extra virgin [20]. Similarly, studies conducted by Guerrini et al. [23] with three oil-borne yeast strains belonging to *N. wickerhamii*, *N. molendinolei*, and *Y. terventina* demonstrated that after six months of storage, the volatile compound content was strongly influenced by the strain of the yeast inoculated. The olive oil samples treated with yeasts showed a higher concentration of compounds responsible for oil defects (trans 2-heptenal, 6-methyl-5-hepten-2-one, 2-octanone) and a lower concentration of C6 volatile carbonyl compounds responsible for positive oil attributes [23].

## 4. Functional Properties of Oil-Borne Yeast Strains

Currently, two yeasts, *S. cerevisiae* and *Saccharomyces boulardii*, which is a strain of the *S. cerevisiae* species, have been recognized as probiotics, and are available on the market. *S. cerevisiae* is frequently isolated from traditional fermented foods, and some strains have shown potential anti-ulcerogenic activity [63]. *S. cerevisiae* is used in the livestock sector, where animal feed is supplemented with the living cells of *S. cerevisiae* to improve growth, health, and immune response in hosts [64]. *S. boulardii*, isolated from the litchi fruit in Indochina by Henri Boulard in the 1920s, is used to treat diarrhea in adults and children infected with *Clostridium difficile*, diarrhea in the human immunodeficiency virus-infected patients, and acute and chronic diarrhea in children and adults [65,66,67]. Other yeast species found in non-oleic habitats include *D. hansenii, Torulaspora delbruecki, Kluyveromyces marxianus, Kazachstania lodderae, C. norvegica,* and *Galactomyces reesii*, and these species have shown tolerance while passing through the gastrointestinal tract and an ability to inhibit enteropathogens [68,69,70]. *K. marxianus* showed an anti-inflammatory activity against an inflammatory bowel disease [71]. Santona et al. [44] reported that among the 64 yeasts isolated from the Sardinian oleic ecosystems, 40 isolates were resistant to pH 2.5 and 55 isolates to 1.5% bile salt. Unlike the *S. boulardii* and *S. cerevisiae* yeasts (which contain only saturated and mono-unsaturated fatty acids), the oil-borne yeast strains showed a higher concentration of polyunsaturated fatty acids (PUFAs) (Table 2) [24]. The high PUFA content in the yeast cells of olive oil, following their autolysis, may be useful in improving the essential fatty acid profile of olive oil, which has low contents of health- beneficial linolenic (omega-3) and linoleic (omega-6) acids [72]. In addition to a PUFA synthesis, other probiotic properties, such as the ability to tolerate unfavorable *in vitro* gastrointestinal conditions, have been demonstrated by yeasts isolated from VOOs. Some of our recent studies showed that unlike the yeasts suspended in the aqueous matrix, the yeasts suspended in oil survived well (100%) during the gastrointestinal digestive tests that were simulated *in vitro* [24]. Based on these results, it may be hypothesized that a majority of yeasts, consumed daily through VOO, reach the intestinal tract. Another probiotic activity, shown by about 50% of the yeasts of the biotic fraction of VOO, is the ability to remove cholesterol *in vitro*. Among the tested yeast strains, the *W. anomalus* species demonstrated the best result (Table 3) [24]. Yeasts of the biotic fraction of VOO also showed antioxidant activity. Twenty-four yeast strains, belonging to eight species isolated from VOO, showed 2,2-diphenyl-1-picryl-hydrazyl (DPPH) free radical-scavenging activity, which was, in some cases, superior to that of the reference probiotic yeast strain *S. boulardii*. The highest antioxidant activity was observed in *N. wickerhamii*, exceeding the activity level of *S. boulardii*. Significantly lower values of antioxidant activity were recorded by *C. adriatica*, *C. diddensiae*, and *Barnettozyma californica* strains. All the oil-borne yeast strains studied *in vitro* showed DPPH free radical-scavenging activity in both physiological solution and olive oil. Tests performed with olive oil enriched with *W. anomalus* and *S. boulardii* yeast biomass showed a positive correlation between the yeast biomass and percentage of antioxidant activity [22].

## 5. Development of a Potentially Functional Olive Oil

The daily intake of the bioactive abiotic endogenous fraction of VOOs, including phenols, depends on genetic, agronomic, and technological factors [73,74,75,76,77]. A wide range of VOOs, containing different amounts of total phenols and having different phenolic compositions, can be found on the market [78]. Different strategies have been proposed to optimize the daily intake of the bioactive abiotic endogenous fraction of VOOs. A strategy to optimize the daily intake of phenolic compounds in the habitual diet is to produce an enriched VOO with well-known bioactive phenols [79,80,81,82,83]. Different sources of natural bioactive ingredients, such as raw materials derived from the same olive tree (mainly leaves or residual olive pomace) obtained after the mechanical extraction of the oil, have been proposed to enrich oils. Other studies used plants and vegetables, mainly herbs and spices [84]. Studies on the enrichment of olive oil, with some components of the VOO biotic fraction, such as yeasts, continue to be rare. So far, research has shown that the biotechnological use of some yeast strains isolated from olive oil has provided scope for further studies. *S. boulardii*, a commercially available probiotic yeast, does not survive for a long time in olive oil [22]. Among the studied oil-borne yeast strains, the *W. anomalus* 2032 yeast strain, which has been characterized by the best performance in removing cholesterol *in vitro* and good antioxidant activity, survived in oil rich in polar phenols [22].

## 6. Conclusions

The biotic fraction of VOO mainly consists of yeasts. To date, approximately twenty-four yeast species have been identified in different types of olive oil, and seven of these species have been classified as new species. The activity of some yeasts of the VOO biotic fraction improves the sensory characteristics of the oil, through the production of β-glucosidase and esterase. Both enzymes act on phenolic compounds that are responsible for the bitter taste of the product. However, yeast can also worsen the quality of the product by allowing the appearance of defects, oxidation of polar phenols, and triacylglycerol hydrolysis. Interesting probiotic activities have been demonstrated *in vitro* by oil-borne yeast species. These activities are associated with a high PUFA content, the ability to remove cholesterol, free radical-scavenging activity, and the ability to colonize in the intestinal tract by overcoming the gastro-pancreatic barrier. The probiotic activity of oil-borne yeast strains may be of importance because VOO enrichment with these yeasts improves the health benefits of the product.

## Figures and Tables

**Table 1:** Yeast species isolated from olive oil identified through D1/D2 (26S) rDNA sequencing.

Yeast Species	Substrate	Location	Reference
*Brettanomyces acidodurans*	olive oil	Spain	[36]
(New species)	spoiled olive oil	Israel	[36]
*Brettanomyces californica*	virgin olive oil	Italy	[22]
*Candida adriatica*	olive oil sediment	Slovenia	[38]
(New species)	virgin olive oil	Italy	[38]
	spoiled olive oil	Croatia	[38]
	oil from decanter	Italy	[23]
*Candida dendronema*	olive oil	Italy	[44]
*Candida diddensiae*	virgin olive oil	Italy	[29]
*Candida norvegica*	virgin olive oil	Italy	[26]
*Candida oleophila*	virgin olive oil	Italy	[26]
*Candida parapsilosis*	virgin olive oil	Italy	[28]
*Candida temnochilae*	olive oil	Italy	[44]
*Debaryomyces hansenii*	virgin olive oil	Italy	[26]
*Groenewaldozyma auringiensis*	olive oil	Spain	[36]
*Lachancea fermentati*	olive oil	Spain	[36]
*Kuraishia capsulata*	virgin olive oil	Italy	[Date unpublished]
*Kuraishia mediterranea*	olive oil sediment	Slovenia	[41]
(New species)	spoiled olive oil	Portugal	[41]
	virgin olive oil	Italy	[Date unpublished]
*Meyerozyma guilliermondii*	virgin olive oil	Italy	[29]
*Nakazawaea molendinolei*	virgin olive oil	Italy	[38]
(New species)	virgin olive oil	Croatia	[38]
	virgin olive oil	Slovenia	[38]
	spoiled olive oil	Israel	[38]
	oil from decanter	Italy	[23]
*Nakazawaea wickerhamii*	virgin olive oil	Italy	[28]
(*Candida wickerhamii*)	oil from decanter	Italy	[23]
*Ogataea histrianica*	olive oil sediment	Slovenia, Italy	[39]
(New species)	virgin olive oil	Italy	[22]
*Ogateae kolombanensis*	olive oil sediment	Slovenia	[39]
(New species)	virgin olive oil	Italy	[Date unpublished]
*Yamadazyma mexicana*	olive oil	Italy	[44]
*Yamadazyma nakazawaea*	olive oil	Italy	[44]
*Saccharomyces cerevisiae*	olive oil	Italy	[44]
*Wickerhamomyces anomalus*	virgin olive oil	Italy	[26]
*Yamadazyma terventina*	virgin olive oil	Italy	[40]
(New species)	oil from decanter	Italy	[23]

**Table 2:** Average fatty acid composition of olive oil-borne yeast species, *Saccharomyces boulardii* and virgin olive oil (%).

Free fatty acid	*Candida adriatica*		*Candida diddensiae*		*Nakazawaea* *molendinolei*		*Nakazawaea* *wickerhamii*		*Wickerhamomyces* *anomalus*		*Yamadazyma* *terventina*		*Saccharomyces* *boulardii*		Olive oil
	No.*	%**	No.	%	No.	%	No.	%	No.	%	No.	%	No.	%	
	2		2		2		1		2		2		1		
Myristic acid (C14:0)		1.02		1.29		0.31		0.22		0.33		0.40		0.89	0.01
Palmitic acid (C16:0)		14.83		20.72		13.35		9.41		18.38		16.34		16.15	12.25
Palmitoleic acid (16:1)		7.56		7.81		9.13		9.52		4.90		8.05		47.01	0.79
Heptadecanoic acid (C17:0)		0.36		0.00		0.64		0.65		0.29		0.49		0.00	0.04
Heptadecenoic acid (C17:1)		2.71		0.98		2.49		4.05		1.98		2.48		0.66	0.06
Stearic acid (C18:0)		2.70		1.72		1.41		0.98		2.46		2.33		5.82	2.61
Oleic acid (C18:1)		36.72		26.91		31.76		32.96		35.47		37.66		26.49	73.50
Linoleic acid (C18:2)		25.51		27.91		31.00		37.31		30.96		25.90		0.00	9.09
Arachic acid (C20:0)		0.09		3.34		3.24		2.05		1.83		0.30		0.00	0.41
Linolenic acid (C18:3)		5.08		9.48		5.97		2.21		3.69		5.07		0.00	0.67
Eicosenoic acid (C20:1)		0.12		0.00		0.00		0.00		0.00		0.03		0.00	0.40
Behenic acid (C22:0)		0.06		0.00		0.00		0.13		0.07		0.10		0.00	0.10
Lignoceric tR acid (C24:0)		2.83		0.32		0.27		0.00		0.20		0.36		2.76	0.04
Total %		99.59		99.76		99.57		99.49		99.58		99.51		99.78	99.96
SFA		21.89		27.39		19.22		13.44		23.51		20.32		25.62	15.72
MUFA		47.11		35.70		43.38		46.53		42.02		48.22		74.16	74.75
PUFA		30.59		36.67		36.97		39.52		34.05		30.97		0.00	9.76

*, number of yeast strains tested; **, % fatty acid composition; SFA, saturated fatty acid; MUFA, mono-unsaturated fatty acid; PUFA, polyunsaturated fatty acid.

**Table 3:** Some in vitro health-related probiotic activities of certain yeast species isolated from olive oil.

Yeast Species.	No.*	Cholesterol Removal (%)	No.	Antioxidant Activity (%)	No.	Specific Activity (U g^−1^ Yeast Biomass)	Reference
*Barnettozyma californica*	0	ND	3	34.17	3	4.44	[22]
*Candida adriatica*	2	14.97	6	47.00	6	6.27	[22,24]
*Candida diddensiae*	2	14.20	4	36.50	4	4.77	[22,24]
*Nakazawaea molendinolei*	2	44.45	3	59.50	3	8.06	[22,24]
*Nakazawaea wickerhamii*	1	39.60	1	83.50	1	11.48	[22,24]
*Ogataea histrianica*	0	ND	1	47.00	1	6.27	[22]
*Yamadazyma terventina*	2	21.45	4	52.13	4	7.00	[22,24]
*Wickerhamomyces anomalus*	2	60.52	2	50.00	2	6.74	[22,24]
Saccharomyces boulardii**	1	35.38	1	70.00	1	9.55	[22,24]

*, number of yeast strains used to produce the average data; U, unit of DPPH radical-scavenging activity that is defined as the antioxidant activity of 1 µg of Trolox; ND, not detected; **, yeast species reference.
